# Gas-Phase Reactions of H_2_CS and H_2_CO with CN: Similarities and Differences from a Computational Study

**DOI:** 10.1021/acsearthspacechem.5c00034

**Published:** 2025-04-14

**Authors:** Silvia Alessandrini, Hexu Ye, Cristina Puzzarini

**Affiliations:** Dipartimento di Chimica “Giacomo Ciamician”, Alma Mater Studiorum - University of Bologna, Via F. Selmi 2, I-40126 Bologna , Italy

**Keywords:** sulfur compounds, thioformyl cyanide, TMC-1, interstellar chemistry, gas-phase kinetics

## Abstract

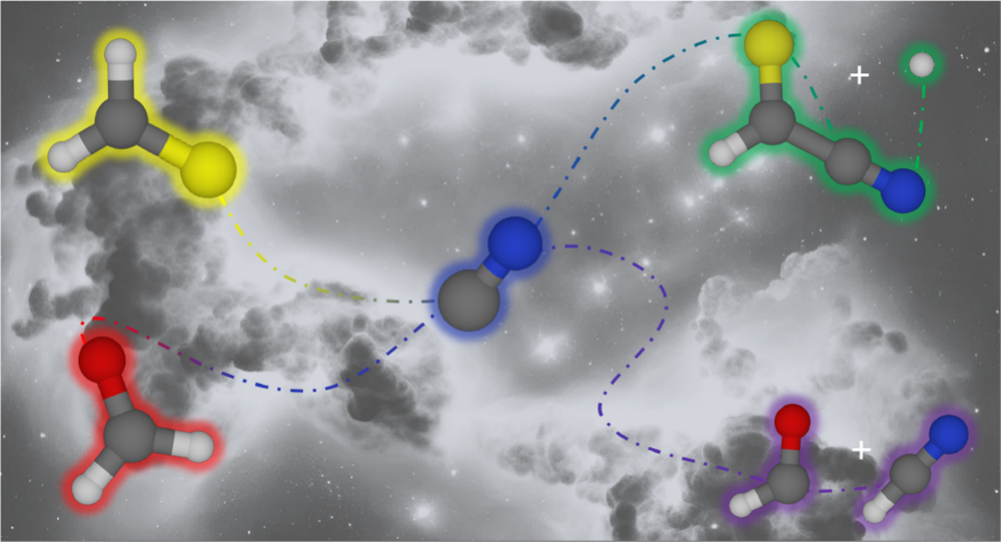

The gas-phase reaction of H_2_CS with the CN radical has been investigated with the aim of understanding its evolution in the interstellar medium (ISM). After the detection of thioformyl cyanide in TMC-1, this process has been incorporated into astrochemical models because it is considered the most efficient (if not the only) gas-phase formation route for such a molecule in dark molecular clouds. However, neither experimental nor theoretical studies have been reported in the literature regarding its feasibility in the typical conditions of the ISM. In this work, the corresponding reactive potential energy surface has been accurately investigated and complemented by ab initio transition state theory calculations to derive global rate coefficients. In view of the potential similarities between the H_2_CS + CN and H_2_CO + CN reactions, this latter process has also been reinvestigated. The availability of experimental data for the H_2_CO + CN reaction has been exploited to derive the accuracy of the computed rate constants for the sulfur-bearing counterpart.

## 1 Introduction

Investigation of reaction pathways involving third-row atoms, like sulfur or phosphorus, is essential to understand how they are incorporated into more complex molecules that might have played a role in the appearance of prebiotic molecules on early Earth. In the case of sulfur, such studies are also relevant to gain insights into the “sulfur depletion problem”,^1,2^ which refers to the difference in sulfur abundance between the diffuse interstellar regions^3,4^ and cold and dense molecular clouds or star-forming regions.^5^ Although it has been suggested that ices could be the most significant reservoir of sulfur in cold regions,^2^ several recent detections of S-bearing species in the TMC-1 dark molecular cloud^6−8^ have questioned this view. While the depletion of sulfur in TMC-1 is still accepted despite the latest detections,^9^ these latter might help in providing some constraints to the sulfur chemistry in cold objects and some clues about the differences with respect to the oxygenated counterparts.

Thirty-three S-bearing molecules have been discovered in TMC-1 so far (for the complete list, the reader is referred to ref (9)). Eighteen out of them have been detected in the last four years: the majority has the [H,C,S] composition, with seven species also bearing nitrogen (namely, NCS, HNCS, HSCN, HCNS, NC_3_S, HC(S)CN, and NCCHCS^10−14^), and only one species containing oxygen (HSO^15^). Formation and destruction pathways for these molecules are mostly missing in the literature. As a consequence, astrochemical models currently fail in reproducing the observed abundances and in providing a reliable sulfur chemistry. Indeed, to overcome the drawback mentioned above, for several processes, rate constants are estimated based on the “oxygen” counterpart.^16^ Aiming at filling this gap, in this work, the gas-phase formation of HC(S)CN, via the reaction between thioformaldehyde (H_2_CS) and the cyano radical (CN), has been investigated. This process is relevant for modeling the sulfur chemistry of the TMC-1 molecular cloud, but it also provides the first insight into the gas-phase reaction mechanisms of thioformaldehyde. In several sources,^17^ TMC-1 being one of them,^11^ this species has nearly the same abundance as formaldehyde (H_2_CO). Therefore, if confirmed that HC(S)CN is efficiently produced, the H_2_CS + CN reaction might help establish its potential presence and/or abundance in other sources, where both reactants are present.

Since sulfur and oxygen belong to the same group of the periodic table, it is interesting to compare the reaction mechanisms of the H_2_CS + CN and H_2_CO + CN gas-phase processes under interstellar conditions in order to highlight similarities and differences. This is particularly relevant in the cases of HC(S)CN and HC(O)CN as potential products because both species have been observed in TMC-1,^11^ with the former being 4 times more abundant than the latter. For formyl cyanide, H_2_CO + CN was considered a plausible gas-phase formation route and was computationally investigated in 2020.^18^ The latter work suggested that the reaction is effective at low temperatures, with a rate coefficient of 1.1 × 10^–9^ cm^3^ molecule^–1^ s^–1^ at 60 K. However, a recent experimental and computational study^19^ pointed out that the main channel for the H_2_CO + CN reaction is instead H abstraction, which leads to the formation of HCN and HCO. Therefore, this reaction was discarded from astrochemical models in favor of the CH_3_CHO + CN one.^16,18^ The reinvestigation of the process involving H_2_CO as a reactant allows the comparison with the experimental results of ref (19), thus permitting one to derive an error estimate for our kinetic outcomes.

Based on the discussion above, in this work, the potential energy surfaces (PESs) of the H_2_CS + CN and H_2_CO + CN gas-phase reactions have been studied to understand which pathways are feasible in the interstellar medium (ISM). This involves analyzing the energy landscape of the reactive systems, thus identifying all possible intermediates and transition states (TSs). Subsequently, kinetic simulations have been carried out in conditions similar to those of the ISM to derive the rate constants for the most important reaction channels. In the following section, computational details are provided. Subsequently, the results are presented and discussed. Finally, concluding remarks are provided.

## 2 Computational Details

Initially, a preliminary investigation of the H_2_CS + CN and H_2_CO + CN PESs has been conducted at the B3LYP^20−22^/jun-cc-pVDZ^23,24^ level of theory including empirical dispersion (D3BJ^25,26^). This level has also been used to carry out intrinsic reaction coordinate calculations^27^ to ensure the correct connection between a given transition state (TS) and the corresponding minima. This first PES investigation has been followed by an improvement in both the structural and energetic characterization as follows.

- The stationary points have been reoptimized using the double-hybrid rev-DSDPBEP86 functional^28^ in conjunction with a partially augmented triple-ζ basis set, i.e., jun-cc-pVTZ,^23,24^ also incorporating the D3BJ empirical correction. Hereafter, this level of theory is shortly denoted as revDSD/junTZ. At each stationary point, the harmonic force field has also been computed at the same level of theory to confirm its nature and to determine the zero-point energy (ZPE) correction within the harmonic approximation.
- On top of the revDSD/junTZ structures, the energy has been improved using the so-called junChS composite scheme.^29,30^
- For critical barriers (i.e., those ruled by TSs lying very close to the reactants’ energy), to ensure a sub-kJ/mol accuracy, the energetics has been reinvestigated employing the HEAT-like approach.^31−33^

While the reader is referred to the cited papers for a detailed description of the junChS and HEAT-like approaches, here, we recall the main features. The former is based on the fc-CCSD(T)/jun-cc-pVTZ level (CCSD(T) stands for coupled-cluster singles and doubles with a perturbative treatment of triple excitations,^34^ and fc stands for frozen-core approximation) and incorporates corrections to account for the extrapolation to the complete basis set (CBS) limit and core–valence (CV) correlation effects at the MP2 level^35^ (Mo̷ller–Plesset second-order theory). The HEAT-like approach is instead a composite scheme entirely based on the coupled-cluster theory: starting from the CCSD(T) method, it accounts for the CBS and CV corrections at the same level and incorporates corrections due to the full treatment of triple and quadruple excitations as well as relativistic and diagonal Born–Oppenheimer corrective terms. In both composite schemes, to avoid spin-contamination of higher electronic spin-states, the restricted open-shell Hartree–Fock wave function has been used as the reference in coupled-cluster and MP2 calculations, as done for previous studies.^30,31^ DFT and junChS computations have been performed with the Gaussian16 (rev. A03) program,^36^ while the CFOUR quantum chemical program package has been employed for the HEAT-like approach.^37,38^ For all calculations involving the sulfur atom, the d-augmented version of the basis sets mentioned above has been used. This is expected to avoid any degradation in accuracy of the computed molecular properties and energetics for molecules containing third-row atoms.

Once the reactive PES is characterized, the next step requires a kinetic investigation in order to understand the most favorite pathways. The theoretical approach for deriving rate constants for a multiwell reaction involves treating each elementary step separately. Transition state theory (TST)^39^ has been employed to calculate the rate constants for the steps involving a TS. Specifically, the microcanonical rate coefficient for a unimolecular elementary step is determined using the Rice–Ramsperger–Kassel–Marcus (RRKM) theory.^40,41^ The RRKM assumption implies that the reactant in each elementary step is in microcanonical equilibrium.

While the treatment above is considered for all of the elementary steps originating from the first bound intermediate, phase space theory (PST)^42,43^ has been used to model the entrance channel. Within PST, the isotropic long-range interaction between the reactants is modeled by an effective *C*_6_*R*^6^ potential, where *R* represents the distance between the two fragments. The coefficient *C*_6_ has been determined by fitting the sampled energies to the function *f*(*R*) = *f*(*R*_0_) – *C*_6_*R*^6^, where *f*(*R*_0_) corresponds to the energy of the reactants at the distance *R*_0_ where no interaction occurs (here, *R*_0_ = 10 Å).

For a multiple-well reaction path, the global rate constant is then derived from the resolution of the master equation, which integrates the bimolecular step treated by PST and the RRKM estimates. Calculations have been performed using the MESS master equation system solver program^44^ at the low-pressure limit and considering a temperature range from 50 to 400 K for H_2_CS + CN and from ∼30 to 800 K for H_2_CO + CN. Subsequently, the temperature dependence of the rate constants has been modeled using the three-parameter Arrhenius–Kooij formula:^45^
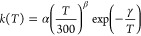
1where α, β, and γ are the fitting parameters: α represents the pre-exponential factor, β accounts for the temperature dependence of the pre-exponential factor, providing a correction for nonstandard reaction dynamics, and γ is related to the activation energy, thus providing the most relevant information on the temperature effect for the rate constant.

## 3 Results and Discussion

In the following, the reactive PESs of the two reactions considered, namely, H_2_CS + CN and H_2_CO + CN, are presented in detail and discussed. Subsequently, for the exothermic and entirely submerged processes, the results of the kinetic investigation are reported.

### 3.1 H_2_CS/H_2_CO + CN PESs

**Figure 1:**
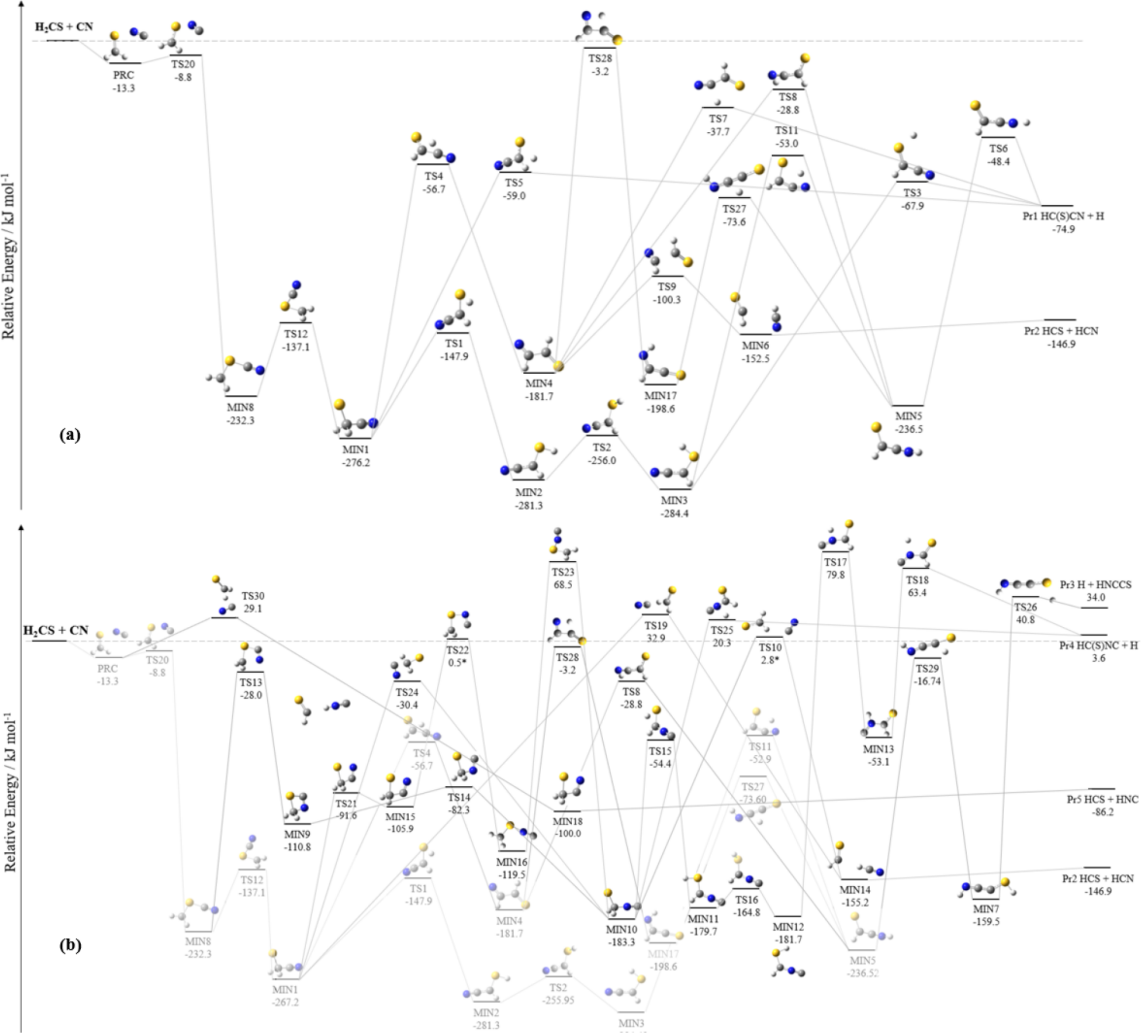
Reaction pathways of H_2_CS + CN: (a) submerged channels and (b) emerged channels (the submerged paths are shadowed). The junChS relative energies, corrected for harmonic revDSD/junTZ ZPE contributions, are reported. * The relative energies of TS22 and TS10 are at the HEAT-like level and corrected for harmonic revDSD/junTZ ZPE.

The reactive PES for the H_2_CS + CN system is depicted in Figure 1, while the relative energies of the corresponding stationary points are summarized in Table 1. This reports the revDSD/junTZ and junChS relative energies, both ZPE-corrected using revDSD/junTZ harmonic frequencies. From the first inspection of this table, it is noted that the differences between the revDSD/junTZ, in the unrestricted formalism, and junChS, based on the ROHF reference wave function, relative energies range approximately between 20 and 40 kJ mol^–1^, thus suggesting that this reactive system might show a non-negligible multireference character as already noted in other studies.^46^ As evident in Figure 1, there are five reaction products: HC(S)CN + H (Pr1), HCN + HCS (Pr2), HNCCS + H (Pr3), HC(S)NC + H (Pr4), and HCS + HNC (Pr5).

**Table 1:** H_2_CS + CN PES: ZPE-Corrected Relative Energies (kJ mol^–1^) of the Stationary Points^a^ (For Labeling, See Figure 1)

	revDSD/junTZ	junChS		revDSD/junTZ	junChS
PRC	–35.8	–13.3	TS9	–110.5	–100.3
MIN1	–310.6	–276.2	TS10^b^	0.06	1.5 (2.8)
MIN2	–305.4	–281.3	TS11	–80.9	–53.0
MIN3	–308.3	–284.4	TS12	–155.8	–137.1
MIN4	–189.0	–181.7	TS13	–32.2	–28.0
MIN5	–267.2	–236.5	TS14	–106.0	–82.3
MIN6	–174.6	–152.5	TS15	–78.9	–54.4
MIN7	–184.0	–159.5	TS16	–191.8	–164.8
MIN8	–263.5	–232.3	TS17	59.1	79.8
MIN9	–137.7	–110.8	TS18	44.4	63.4
MIN10	–216.4	–183.3	TS19	2.4	32.9
MIN11	–205.8	–179.7	TS20	–35.0	–8.8
MIN12	–207.5	–181.7	TS21	–101.3	–91.6
MIN13	–80.2	–53.1	TS22^b^	–20.7	1.7 (0.5)
MIN14	–178.4	–155.2	TS23	49.2	68.5
MIN15	–113.4	–105.9	TS24	–62.1	–30.4
MIN16	–148.1	–119.5	TS25	2.9	20.3
MIN17	–216.6	–198.6	TS26	30.8	40.8
MIN18	121.8	–100.0	TS27	–104.3	–73.6
TS1	–170.5	–147.9	TS28	–5.1	–3.2
TS2	–279.1	–256.0	TS29	–39.2	–16.8
TS3	–83.6	–67.9	TS30	8.1	29.10
TS4	–69.1	–56.7	Pr1	–106.4	–74.9
TS5	–73.7	–59.1	Pr2	–168.1	–146.9
TS6	–58.6	–48.4	Pr3	29.3	34.0
TS7	–47.1	–37.7	Pr4	5.9	3.6
TS8	–48.7	–28.8	Pr5	–104.8	–86.2

a Harmonic ZPE corrections are at the revDSD/junTZ level of theory. The ZPE-corrected absolute energy of H_2_CS is −437.079180 *E*_h_ at the revDSD/junTZ level and −437.308447 *E*_h_ at the junChS level. The ZPE-corrected absolute energy of CN is −92.569583 *E*_h_ at the revDSD/junTZ level and −92.701422 *E*_h_ at the junChS level.

b In parentheses, the ZPE-corrected HEAT-like relative energies are given.

Figure 1 consists of two panels, both reporting the ZPE-corrected junChS relative energies, which are those discussed in the following. In panel (a), the submerged reaction pathways are depicted. Conversely, panel (b) reports the formation of Pr3, Pr4, and Pr5, which are endothermic products (Pr3 and Pr4), and show pathways with emerged barriers with respect to the reactants. As evident from both panels, the initial step of the reaction is the formation of a weak interaction complex (PRC), with a relative energy of −13.3 kJ mol^–1^. This then evolves to MIN8 (−232.3 kJ mol^–1^) via TS20 (−8.8 kJ mol^–1^). In both panels, MIN8 subsequently leads to MIN1 (−276.2 kJ mol^–1^) via TS12 (−137.2 kJ mol^–1^). Focusing on panel (a), Pr1 can be formed directly from MIN1 through a one-step reaction via TS5 (−59.1 kJ mol^–1^). Additionally, hydrogen migration between carbon atoms in MIN1 results in the formation of MIN4, with a relative energy of −181.7 kJ mol^–1^, which can further proceed toward the formation of Pr1 via TS7 (−37.7 kJ mol^–1^). An alternative pathway for forming Pr1 from MIN4 involves a series of steps: MIN4 → TS28 (−3.2 kJ mol^–1^) → MIN17 (−198.6 kJ mol^–1^) → TS27 (−73.6 kJ mol^–1^) → MIN5 (−236.5 kJ mol^–1^) → TS6 (−48.4 kJ mol^–1^) → Pr1. Additionally, MIN4 is connected to MIN5 also via TS8 (−28.8 kJ mol^–1^). Furthermore, the cleavage of the carbon–carbon bond in MIN4 via TS9 (−100.3 kJ mol^–1^) results in the formation of MIN6, which lies 152.5 kJ mol^–1^ below the reactants. From MIN6, Pr2 (HCS + HCN) is formed without overcoming any additional energy barrier. In addition to the pathways discussed above, MIN1 can also form MIN2 through a hydrogen migration step via TS1 (−147.9 kJ mol^–1^). Subsequently, MIN2 interconverts to MIN3 (−284.4 kJ mol^–1^) by overcoming a relatively small energy barrier ruled by TS2 (−256.0 kJ mol^–1^). MIN3 is also connected to MIN5 via TS11, which is located 53.0 kJ mol^–1^ below the energy of the reactants. Additionally, MIN3 can directly evolve into Pr1 via TS3 (−67.9 kJ mol^–1^). This intricate network of reaction pathways provides a comprehensive understanding of how complex the mechanisms involved in the reactivity of H_2_CS with CN are, highlighting multiple potential routes for the formation of HC(S)CN + H and HCS + HCN.

From an inspection of panel (b) of Figure 1, we note that the formation of Pr3, Pr4, and Pr5 is precluded in the ISM conditions because of the endothermicity of the corresponding channels and/or the presence of emerged barriers. In this panel, we also find two pathways leading to Pr2, which, according to junChS energetics, should be excluded from further investigation because one is ruled by TS19, which lies at 32.9 kJ mol^–1^ above the reactants, and the other by TS10, which instead emerges by only 1.5 kJ mol^–1^. Since this value is smaller than the typical accuracy of junChS energetics,^30,47^ TS10 and the corresponding barrier have been reinvestigated at a higher level of theory, i.e., using the HEAT-like approach. This not only confirmed that TS10 is emerged (+2.8 kJ mol^–1^), but it even suggested an increased energy with respect to reactants.

**Figure 2:**
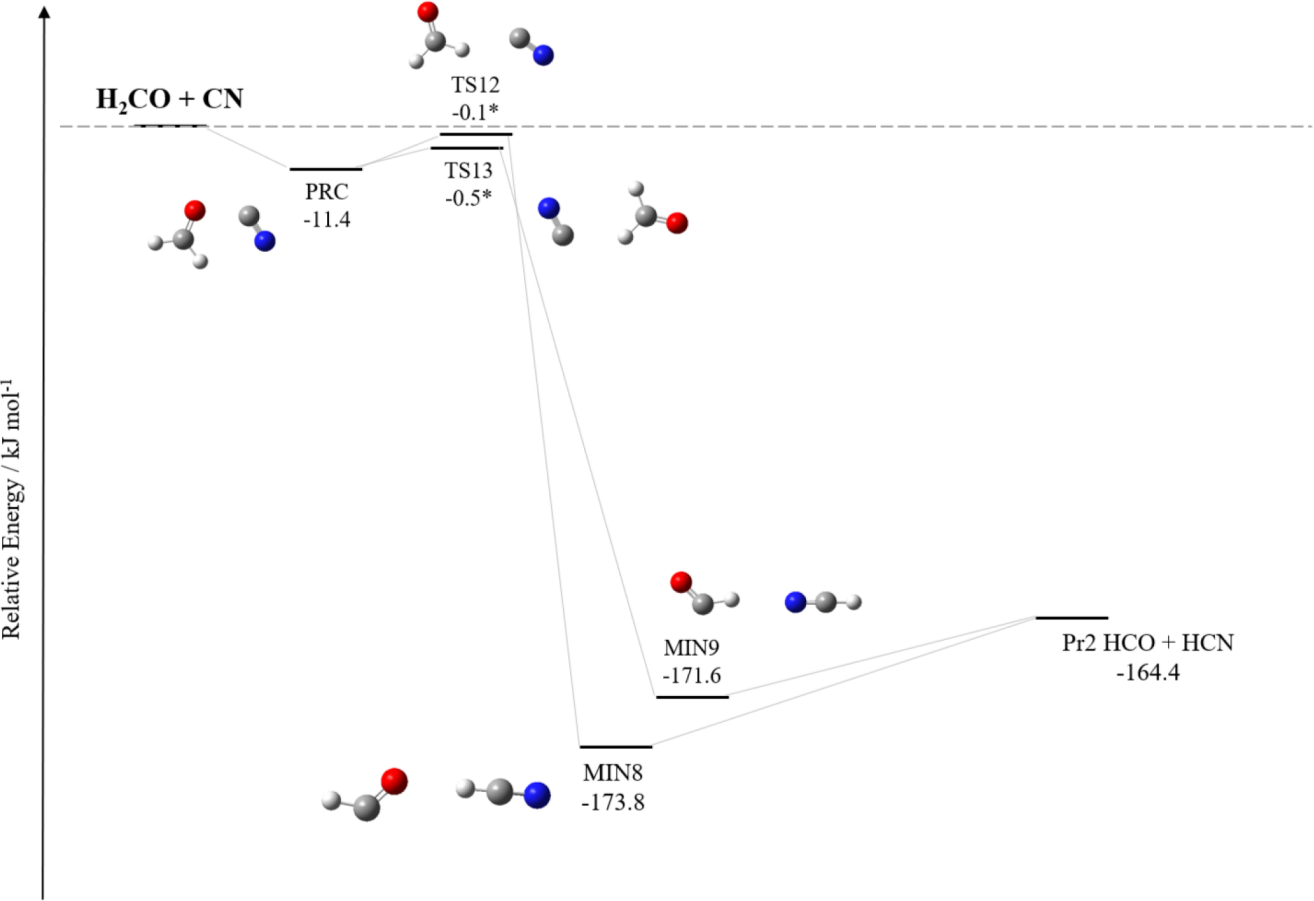
Reaction pathways of H_2_CO + CN: submerged channels. The junChS relative energies, corrected for harmonic revDSD/junTZ ZPE contributions, are reported. *The relative energies of TS12 and TS13 are at the HEAT-like level and corrected for harmonic revDSD/junTZ ZPE.

**Figure 3:**
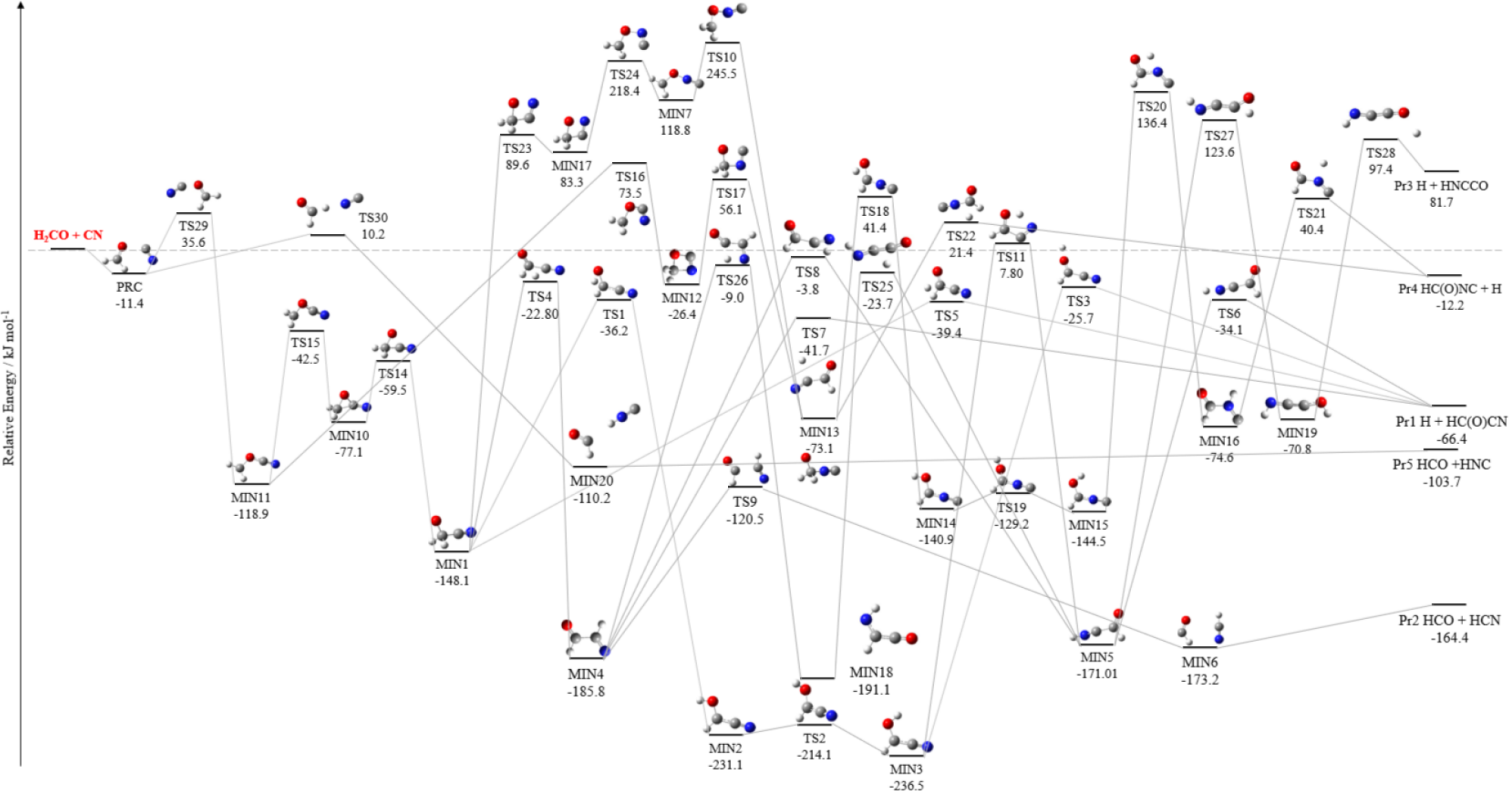
Reaction pathways of H_2_CO + CN: emerged channels. The junChS relative energies, corrected for harmonic revDSD/junTZ ZPE contributions, are reported.

As mentioned in the Introduction section, the H_2_CO + CN reaction has been reinvestigated with the aim of providing an error estimate for the H_2_CS + CN reaction. The corresponding pathways are illustrated in Figures 2 and 3, with the ZPE-corrected relative energies of the stationary points being summarized in Table 2. As above, in both figures, the relative energies are at the junChS level, on top of the revDSD/junTZ geometries, and incorporate harmonic ZPE corrections at the same level. Figure 2 only reports the submerged pathways, while Figure 3 shows those channels presenting at least one emerged TS. Concerning the overall reactions, only the process leading to Pr3 (HNCCO + H) is endothermic, while all of the other products, namely, Pr1 (HC(O)CN + H), Pr2 (HCO + HCN), Pr4 (HC(O)NC + CN), and Pr5 (HCO + HNC), result from exothermic reactions. However, as evident from the inspection of Figures 2 and 3, only Pr2 can be formed from an entirely submerged path. All processes start from the formation of a weak interaction complex, denoted as PRC (−11.4 kJ mol^–1^), which leads to MIN8, MIN9, and MIN11. As shown in Figure 3, from MIN1, the formation of HC(O)CN + H proceeds through different pathways, most of them being submerged. However, MIN1 is formed from MIN11 (via the submerged TS14 and TS15), which in turn originates from PRC via TS29, involving a barrier that emerges by about 35 kJ mol^–1^.

**Table 2:** H_2_CO + CN PES: ZPE-Corrected Relative Energies (kJ mol^–1^) of the Stationary Points^a^ (For Labeling, See Figures 2 and 3)

	revDSD/junTZ	junChS		revDSD/junTZ	junChS
PRC	–25.4	–11.4	TS8	–27.5	–3.8
MIN1	–178.3	–148.4	TS9	–135.3	–120.5
MIN2	–248.9	–231.1	TS10	229.3	245.5
MIN3	–254.3	–236.5	TS11	–12.4	7.8
MIN4	–203.1	–185.8	TS12^b^	–2.1	–0.002 (−0.1)
MIN5	–192.9	–171.1	TS13^b^	–1.7	0.6 (−0.5)
MIN6	–197.6	–173.2	TS14	–73.1	–59.5
MIN7	91.5	118.8	TS15	–60.0	–42.5
MIN8	–198.1	–173.2	TS16	78.0	73.5
MIN9	–196.8	–171.6	TS17	41.7	56.1
MIN10	–102.4	–77.1	TS18	18.8	41.4
MIN11	–147.8	–118.9	TS19	–149.9	–129.2
MIN12	–50.8	–26.4	TS20	87.7	136.4
MIN13	–101.3	–73.1	TS21	19.4	40.4
MIN14	–160.2	–140.9	TS22	1.9	21.4
MIN15	–164.1	–144.5	TS23	91.0	89.6
MIN16	–101.4	–74.6	TS24	199.9	218.4
MIN17	82.9	83.0	TS25	–50.7	–23.6
MIN18	–213.3	–191.0	TS26	–21.9	–9.0
MIN19	–86.9	–70.8	TS27	98.3	123.6
MIN20	–133.1	–110.2	TS28	80.3	97.4
TS1	–57.9	–36.2	TS29	18.4	35.6
TS2	–231.8	–214.1	TS30	11.0	10.2
TS3	–42.7	–25.7	Pr1	–96.8	–66.4
TS4	–36.6	–22.8	Pr2	–189.2	–164.4
TS5	–58.9	–39.4	Pr3	53.4	81.7
TS6	–55.2	–34.1	Pr4	–40.2	–12.2
TS7	–59.9	–41.7	Pr5	125.3	103.7

a Harmonic ZPE corrections are calculated at the revDSD/junTZ level of theory. The ZPE-corrected absolute energy of H_2_CO is −114.328774 *E*_h_ at the revDSD/junTZ level and −114.468748 *E*_h_ at the junChS level. The ZPE-corrected absolute energy of CN is −92.569583 *E*_h_ at the revDSD/junTZ level and −92.701422 *E*_h_ at the junChS level.

b In parentheses, the ZPE-corrected HEAT-like relative energies are given.

The only open paths in the interstellar conditions (Figure 2) are those leading to MIN8 (−173.2 kJ mol^–1^) and MIN9 (−171.6 kJ mol^–1^), via TS12 and TS13, respectively. Both intermediates then dissociate into Pr2 without overcoming any barrier. However, TS12 and TS13 have energies close to that of the reactants once the ZPE correction is incorporated. If revDSD/junTZ energies are used, TS12 and TS13 lie 2.1 and 1.7 kJ mol^–1^ below the reactants, respectively. The corresponding relative ZPE-corrected junChS energies are slightly higher: −0.002 and +0.6 kJ mol^–1^, respectively. To clarify whether these TSs are submerged or not, the very accurate HEAT-like approach has been employed. According to it, TS12 and TS13 lie 0.1 and 0.5 kJ mol^–1^ below the energy of the reactants (ZPE-incorporated), respectively, thus confirming that these barriers are indeed submerged. Therefore, the H_2_CO + CN reaction leads to Pr2 (HCO + HCN) under ISM conditions. These findings are in line with the results reported in ref (19), thus confirming that the H_2_CO + CN reaction is not a primary formation route for interstellar formyl cyanide.

From a careful inspection of Figures 1–3, we note that the intermediate MIN1 is a sort of turning point. Starting from it, all pathways toward the products originate and a general, common mechanism for the two reactions can be drawn. The portion of the reactive PES preceding MIN1 can be seen as a blow-up on the entrance channels. In both reactions, we observed the initial formation of PRC, which evolves—in a different number of steps—into MIN1. However, here, the reactive PESs differ significantly. In fact, in the rearrangements from PRC to MIN1, all of the barriers are submerged for H_2_CS + CN, while that ruled by TS29 is emerged for H_2_CO + CN. This difference leads to completely different outcomes in the extreme conditions of the ISM. The major difference observed on the H_2_CO + CN and H_2_CS + CN PESs can be traced back to TS29 for H_2_CO + CN and TS20 for H_2_CS + CN. In both cases, the TS is the one connecting PRC to the first stable intermediate (MIN11 for H_2_CO + CN and MIN8 for H_2_CS + CN), providing access to the channel(s) of the radical addition, which results in the formation of Pr1. In the case of H_2_CS + CN, the TS20 lies about 9 kJ mol^–1^ below the reactants, while the TS29 of the H_2_CO + CN reaction is located 36 kJ mol^–1^ above the reactants. The comparison of the TS geometries allows us to draw some interesting conclusions. It is noted that, in TS20 (H_2_CS + CN), the sulfur atom interacts with the π orbital of CN (which lies out of the H_2_CS plane), while TS29 (H_2_CO + CN) is characterized by an in-plane O–CN interaction. These geometries clearly point out that the different electronegativity and polarizability of the sulfur and oxygen atoms lead to interactions that are different in nature, with that in TS20 (H_2_CS + CN) stabilizing the adduct more than that occurring in TS29.

Before moving to the kinetic considerations, it is interesting to note that, according to our PES, both the HCS + HCN and the HCS + HNC reactions cannot lead to the formation of HC(S)CN + H because the processes are endothermic.

### 3.2 Kinetic Simulations

The kinetic investigation has been conducted in order to determine the actual feasibility of the reactions discussed above, thus deriving their potential impact on astrochemical models. Global rate constants have been computed as explained in the computational details section for those pathways that are thermodynamically accessible in the ISM.

The barrierless entrance channel has been modeled using PST, which requires the derivation of the *C*_6_ coefficient of the *R*^–6^ function modeling the potential energy curve connecting PRC to the reactants. For both H_2_CS + CN and H_2_CO + CN, these curves are shown in the Supporting Information (SI). For the former reaction, the *R* coordinate corresponds to the distance between the nitrogen atom of the CN radical and the sulfur atom of H_2_CS, with *R* ranging from 2.2 to 10 Å, and a *C*_6_ value of 149.37 *a*_0_^6^*E*_h_ has been obtained. For H_2_CO + CN, *R* indicates the distance between the carbon end of the CN radical and the oxygen atom of H_2_CO, with *R* ranging from 2.1 to 10 Å, and the derived *C*_6_ value is 170.83 *a*_0_^6^*E*_h_. As mentioned, once the unimolecular adduct is formed, the rate constants of all of the elementary steps have been calculated using TST, accounting for tunneling through the Eckart model^48^ and using the rigid-rotor harmonic-oscillator approximation for all of the stationary points. Although the Eckart model is a simple approximation, the differences with respect to the small-curvature approximation^49^ are expected to fall within the error committed by using PST. In addition, the Eckart model is largely used in the investigation of astrochemical reactions.^19,50^ Lastly, the global rate constants of all multiwell pathways have been evaluated by solving the master equation, which integrates the bimolecular step treated by PST and the RRKM estimates. These calculations have been performed at the zero-pressure limit in the 50–400 K range for the H_2_CS + CN system, while the temperature has been varied from 32 to 800 K for H_2_CO + CN. A pressure value of 1 × 10^–12^ atm has been assumed as the low-pressure limit for both reactions for the simulations. The pressure dependence (reported in the SI) shows that only the channel leading to HCS + HCN seems to have a very small pressure dependence for *p* > 0.01 atm.

**Figure 4:**
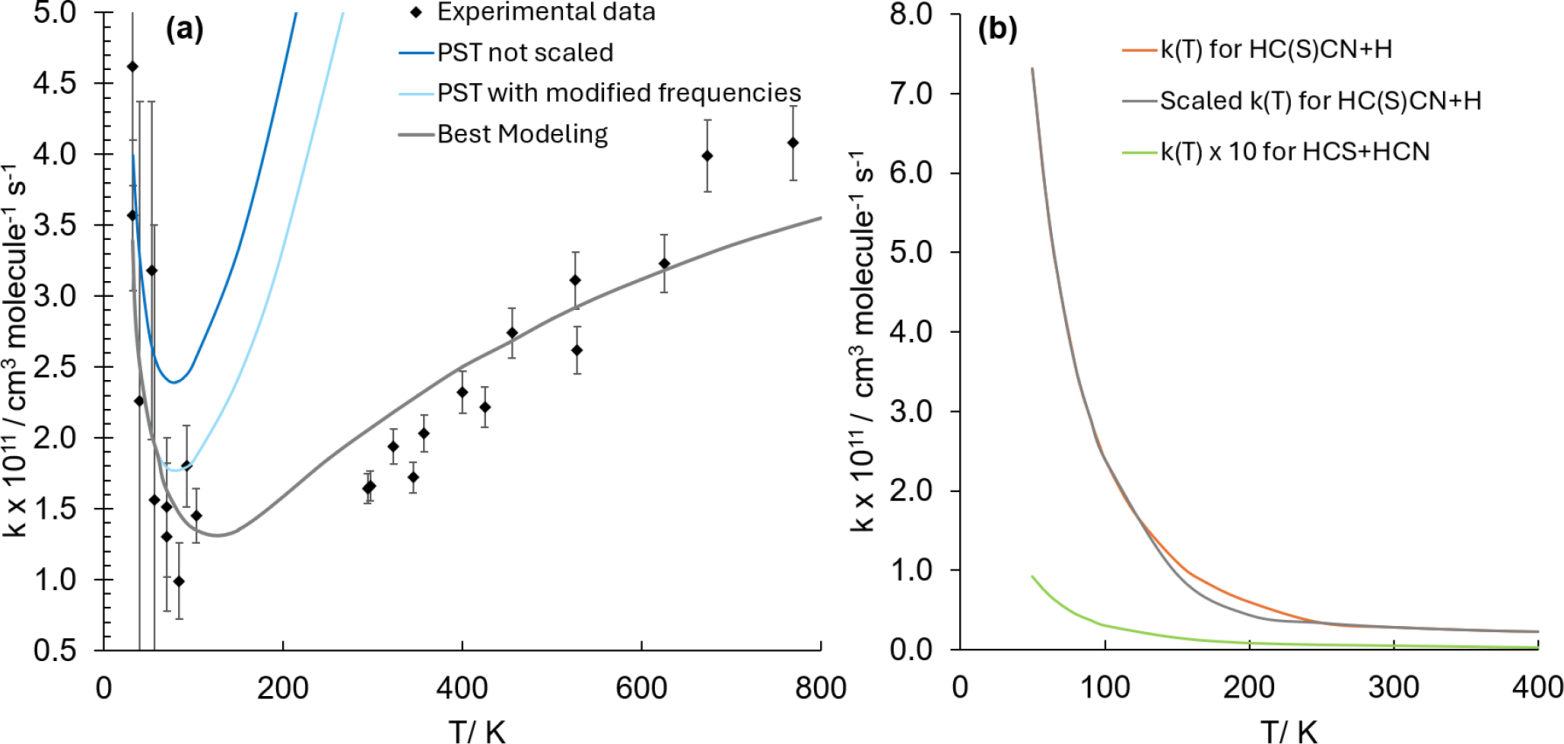
Panel (a): Rate constants computed in the 32–800 K range at a pressure of 1 × 10^–12^ atm for the H_2_CO + CN → HCO + CN reaction. The blue curve represents the simulation obtained from the computed data, while the light-blue one is the simulation with the lowest vibrational frequencies of the TS12 and TS13 modified (see text). The gray line represents the best modeling, as explained in the text. Panel (b): Rate constants computed in the 50–400 K range at a pressure of 1 × 10^–12^ atm for the formation of HC(S)CN (orange curve) and HCS + HCN (green curve). The gray curve results from the scaled rate constants based on the error estimate derived from the comparison with the experiment for the H_2_CO + CN reaction (see text for details).

The H_2_CO + CN reaction has been studied experimentally in different temperature ranges;^19,51,52^ thus, the present simulations can be compared with the available experimental data in order to derive an estimate of the error affecting the kinetic simulations for the H_2_CS + CN reaction. Indeed, the PST employed for describing the entrance channel is expected to provide an overestimation of the barrierless rate. For H_2_CO + CN, as mentioned above, such an overestimation can be evaluated owing to the availability of experimental data at different temperatures. In panel (a) of Figure 4, the comparison of our computed rate constants with the experimental data, in the 32–800 K range, is reported. Experiments were performed using a Laval nozzle apparatus combined with laser-induced fluorescence (LIF) detection in the 32–103 K range^19^ and pulsed laser photolysis–laser-induced fluorescence (PLP–LIF) technique in the 294–769 K range.^51,52^ In the present master equation simulations, the same collision model of ref (19) has been employed. Similarly, the experimental error associated with the data between 300 and 800 K was taken from ref (19). Back to panel (a) of Figure 4, the blue line is the simulation based on our computed rate constants. At all temperatures, a clear overestimate of the experimental rates is noted. By correcting the lowest harmonic frequency of TS12 and TS13 (24 and 17 cm^–1^, respectively) by 10 cm^–1^, the light-blue curve is derived. The correction applied is in line with the typical underestimation of these low vibrational frequencies. Such a correction is surely an arbitrary one, but it lies within the computational uncertainty expected for such low harmonic frequencies.^53^ It is evident that the light-blue simulation reproduces reasonably well the rate coefficients at temperatures below 100 K. Starting from the incorporation of this correction, the overestimation in the description of the entrance channel by the PST can be evaluated. Based on a fit of the experimental data, the entrance channel was found to be overestimated by a factor of 15 in the 150–800 K range and by about 9 at *T* = 100 K. However, for temperatures below 80 K, the entrance channel does not seem to be overestimated. Using the corrective factors mentioned above, the “Best-Modeling” plot of Figure 4a has been derived. Even if our corrected model is not as sophisticated as the one derived in ref (19) (which should be considered the reference for this reaction), the rates follow the correct trend.

Moving to the H_2_CS + CN reaction, we computed the rate constants for the pathways leading to Pr1 (HC(S)CN + H) and Pr2 (HCS + HCN). The results are graphically shown in Figure 4, panel (b). From this figure, it is evident that the rate constants increase as the temperature decreases, with the steepening becoming particularly pronounced at temperatures below 150 K. The formation of Pr1 (orange line) shows the largest rate constants, with those leading to Pr2 being about 1–2 orders of magnitude smaller. At *T* = 50 K, the rate coefficient for the H_2_CS + CN → Pr1 reaction is 7.31 × 10^–11^ cm^3^ molecule^–1^ s^–1^, while that for the process leading to Pr2 is 9.26 × 10^–13^ cm^3^ molecule^–1^ s^–1^. The formation of Pr1 is favored because it involves four possible pathways, one of which is a two-step mechanism with the lowest energy barrier. Since we have derived an overestimation factor of 15 for the H_2_CO + CN reaction at *T* > 150 K, we have applied the same factor to the rate coefficients of the H_2_CS + CN → Pr1 reaction. The resulting simulation is shown in panel (b) of Figure 4 using a gray line.

The temperature dependence of rate constants for the H_2_CS + CN and H_2_CO + CN reactions has been modeled using the Arrhenius–Kooij equation and employing the computed data in the 50–100 K range for the H_2_CS + CN reaction and those in the 30–100 K interval for H_2_CO + CN. The corresponding parameters are collected in Table 3 and can be used to extrapolate the rate coefficients at temperatures outside the range considered. In particular, for the H_2_CS + CN → Pr1 reaction, we expect a rate of 1.3 ± 0.03 × 10^–10^ cm^3^ molecule^–1^ s^–1^ at *T* = 30 K. This value is somewhat in agreement with the assumption made in ref (16) (UMIST database), where a rate coefficient of 1 × 10^–10^ cm^3^ molecule^–1^ s^–1^ is assumed and no temperature dependence is considered.

**Table 3:** Arrhenius–Kooij Parameters for the H_2_CS + CN and H_2_CO + CN Reactions^a^

*T* range	product	α/cm^3^ molecule^–1^ s^–1^	β	γ/K	rms of the fit^b^
20–100	Pr2, HCO + HCN	(9.12 ± 0.71) × 10^–12^	–0.28 ± 0.10	–21.96 ± 5.2	6.55 × 10^–13^
20–200	Pr1, HC(S)CN + H	(3.30 ± 0.02) × 10^–12^	–2.15 ± 0.01	38.1 ± 0.45	2.6 × 10^–14^
20–200	Pr2, HCS + HCN	(4.20 ± 0.01) × 10^–14^	–2.14 ± 0.01	37.3 ± 0.44	3.04 × 10^–16^

a The fit employed the ZPE-corrected junChS relative energies (Table 1).

b rms stands for root-mean-square deviation of the fit.

## 4 Conclusions

The present work investigated the reaction mechanisms of the gas-phase processes involving two isoelectronic molecules, H_2_CO and H_2_CS, and the CN radical under interstellar conditions. Here, we recall that only exothermic reactions showing submerged barriers can occur at the typical low temperatures of the ISM, with the only possible exceptions resulting from quantum tunneling effects.^54^ While the focus of our study was mainly on the first characterization of the gas-phase H_2_CS + CN reaction, the experimental results available for H_2_CO + CN allowed us to assess the accuracy of our methodology. The good agreement at temperatures below 150 K suggests that the rate constants obtained for the H_2_CS + CN reaction pathways leading to HC(S)CN + H (Pr1) and to HCS + HCN (Pr2) at the low temperatures of interest in the ISM are reliable and rather accurate.

The investigation of the H_2_CO + CN and H_2_CS + CN reactive systems pointed out significant differences in the potential energy landscape that can contribute to explaining why HC(S)CN is more abundant than HC(O)CN in TMC-1. While H_2_CO + CN leads to HCO + HCN because of an emerged barrier in the entrance channels, HC(S)CN is the kinetically most favorite product of the H_2_CS + CN reaction despite being thermodynamically less stable than HCS + HCN. Furthermore, we also pointed out that simple bimolecular reactions like HCS + HCN or HCS + HNC cannot lead to the formation of HC(S)CN + H because the corresponding reactions are endothermic.

To conclude, the H_2_CS/H_2_CO + CN reactions provide an interesting example to demonstrate that the rate coefficients of oxygenated compounds cannot be safely employed for the reactivity of the corresponding sulfur counterparts. Furthermore, it well illustrates that kinetic simulations are needed to provide the final answer on the feasibility and effectiveness of reaction pathways at the low temperatures characteristic of dark molecular clouds.

## Supporting Information Available

Barrierless entrance channel for H_2_CS and H_2_CO reacting with the CN radical; and pressure dependence of the H_2_CS + CN rate coefficients at 100 K (PDF)
