# Supplementary material for: Gas-Phase Reactions of H2CS and H2CO with CN: Similarities and Differences from a Computational Study
Source: ACS Earth Space Chem. 2025 Apr 14;9(5):1217–26. doi: 10.1021/acsearthspacechem.5c00034 (PMC12086962; doi:10.1021/acsearthspacechem.5c00034)
Supplement: Supplementary file 1 — sp5c00034_si_001.pdf [file sp5c00034_si_001.pdf]

Gas-phase Reactions of H_2CS and H_2CO with CN: Similarities and Differences from a Computational Study

Silvia Alessandrini,* Hexu Ye, and Cristina Puzzarini*

Dipartimento di Chimica "Giacomo Ciamician", Alma Mater Studiorum - University of Bologna, Via F. Selmi 2, Bologna, I-40126, Italy

E-mail: silvia.alessandrini7@unibo.it; cristina.puzzarini@unibo.it

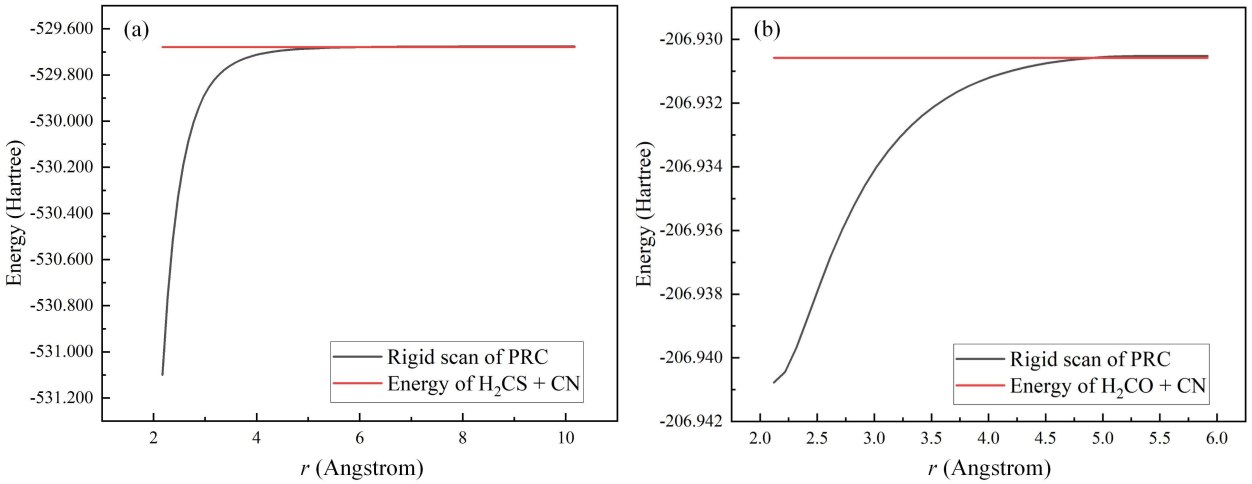

Figure S1: Potential energy curves for the entrance channel of H_2CS/H_2CO + CN. The black lines represent the revDSD/junTZ rigid scan. The energy of the separated fragments (in red) is also provided.

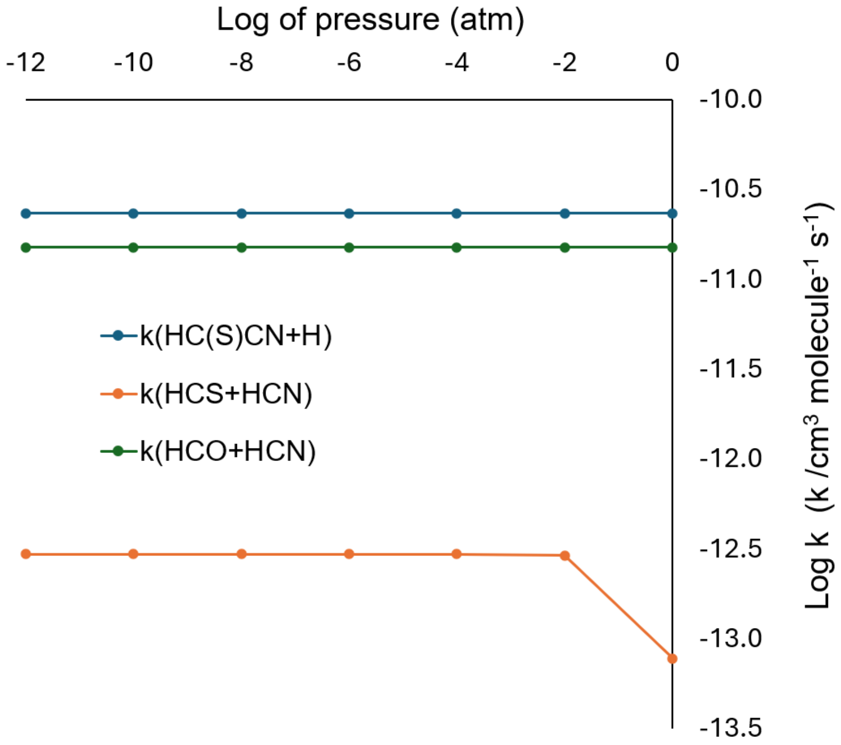

Figure S2: Pressure dependence of the global rate constants for the H_2CS + CN → Pr1 and H_2CS + CN → Pr2 reactions and for H_2CO + CN → Pr2.
